# Cell-Free DNA-Based Blood Tests for Colorectal Cancer Screening: Current Advances, Challenges, and Future Perspectives

**DOI:** 10.3390/diagnostics16203336

**Published:** 2026-10-14

**Authors:** Ghada Alkhawlani, Nasser Alsanea, Reem Alharbi, Meshal Al-sharafa, Sami Alonazi, Samaa Abdullah, Mariam M. Aleissa, Sameera Abuaish, Hanaa A. Yamani, Alaa A. Al-Masud

**Affiliations:** 1 Nourah’s Tissue Biobank, Natural and Health Science Research Center, Princess Nourah bint Abdulrahman University, Riyadh 11671, Saudi Arabia; ghadaalkawlani@gmail.com (G.A.); shalonazi@pnu.edu.sa (S.A.); 2 Department of Biology, College of Science, Princess Nourah bint Abdulrahman University, Riyadh 11671, Saudi Arabia; hayamani@uj.edu.sa; 3 Surgery Department, College of Medicine, Princess Nourah bint Abdulrahman University, Riyadh 11671, Saudi Arabia; nalsanea@pnu.edu.sa; 4 College of Medicine, Princess Nourah bint Abdulrahman University, King Abdullah Bin Abdulaziz University Hospital, Riyadh 11564, Saudi Arabia; reaalharbi@kaauh.edu.sa; 5 Faculty of Pharmacy, Applied Science Private University, Amman 11931, Jordan; 6 Public Health Lab, Public Health Authority, Riyadh 12382, Saudi Arabia; mariam_al_eissa@hotmail.com; 7 Basic Sciences Department, College of Medicine, Princess Nourah bint Abdulrahman University, Riyadh 11671, Saudi Arabia; syabuaish@pnu.edu.sa; 8 Department of Biological Sciences, College of Science, University of Jeddah, Jeddah 21589, Saudi Arabia

**Keywords:** cell-free DNA, colorectal cancer, early detection, fragmentomics, multi-omics

## Abstract

**Background**: Colorectal cancer (CRC) is a major cause of rising global rates of cancer-related morbidity and mortality. CRC rates are particularly increasing among younger populations; however, the implementation of effective CRC screening programs remains limited. Colonoscopy, the gold standard CRC screening technique, is restricted by complex procedures, invasive methods, and low patient adherence. Therefore, non-invasive screening through circulating cell-free DNA (cfDNA)-based blood tests is emerging as an effective alternative. **Methods**: This narrative review summarizes recent advances in cfDNA-based blood tests for colorectal cancer screening and early detection. Relevant English-language publications from 2021 to 2025 were searched on PubMed, ScienceDirect, and Google Scholar databases, supplemented by examination of reference lists. The study evaluated various diagnostic performance measures [sensitivity, specificity, and AUC (area under the curve)] and compared cfDNA-based approaches with previously established screening methods. **Results**: CRC detection and diagnostic efficacies of cfDNA-based technologies (mutation analysis, fragmentomics, integrated multi-omics approaches, and methylation profiling) have been reported in several recent studies. Fragmentomics and integrated multi-omics strategies have reported high diagnostic accuracy in a small number of heterogeneous, non-pooled studies. During a large-scale ECLIPSE study, involving an average-risk screening population, the Shield blood-based assay demonstrated 83.1% sensitivity for CRC, whereas the sensitivity for advanced precancerous lesions was only 13.2%. However, lack of assay standardization, low sensitivity for advanced precancerous lesions, methodological heterogeneity, and the need for prospective validation before routine clinical implementation remain major limitations. **Conclusions**: cfDNA-based non-invasive screening tools offer promising colorectal cancer detection accuracy. However, prospective validation, standardization, and optimization of analytical workflows are mandatory for the widespread clinical implementation of these techniques.

## 1. Introduction

Colorectal cancer (CRC) is a major global malignancy, ranking third in incidence and second in cancer-related mortalities [1]. Approximately 1.9 million new CRC cases and 904,000 deaths were reported in 2022 [2]. CRC incidence has been projected to reach 3.2 million annual cases and 1.6 million deaths by 2040 [3]. Recent global analyses indicate a substantial and increasing CRC burden and support the optimization of screening programs [4].

**Figure 1:**
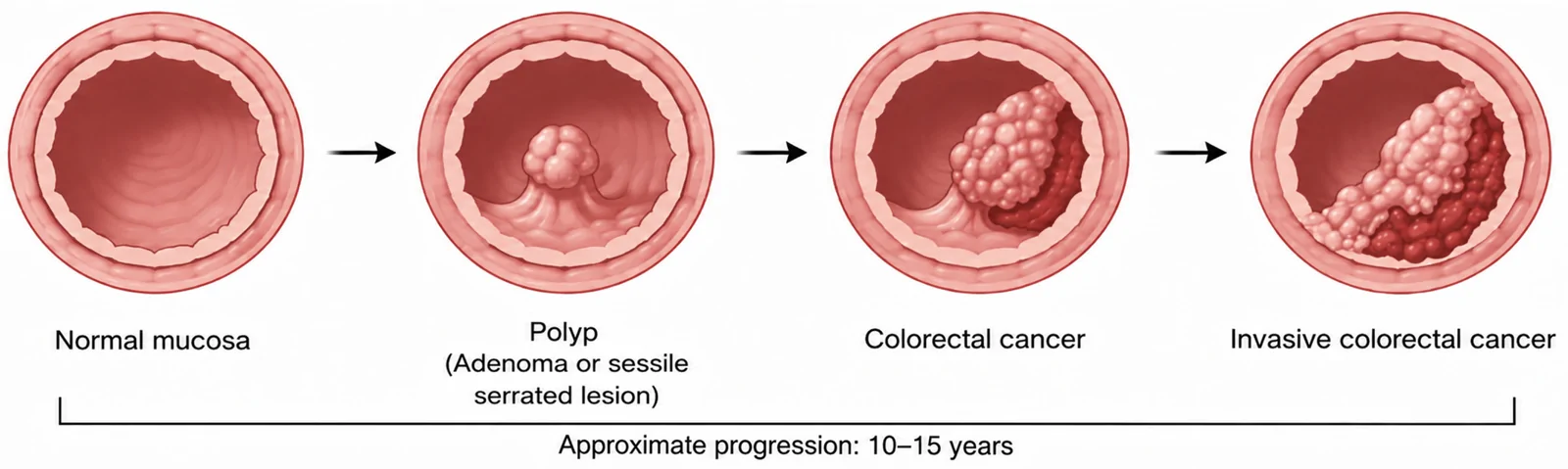
Schematic representation of the progression of colorectal carcinogenesis from normal colonic mucosa through precursor polyps to colorectal cancer and invasive carcinoma. The adenoma–carcinoma sequence is estimated to develop over an average period of 10–15 years [7]. The figure was created by the authors based on published literature [9]. A complex interplay of multiple factors (environmental, genetic, and epigenetic) contributes to CRC development. These factors involve key processes like cell proliferation, angiogenesis, invasion, and metastasis [10,11]. Gene mutations (*PIK3CA*, *APC*, *TP53*, and *KRAS*) and epigenetic alterations (genomic instability and DNA methylation) trigger tumor development and progression [12].

CRC was traditionally associated with older adults; however, its incidence has increased among individuals below 50 years of age [5]. Notably, a significant rise has also been reported among individuals aged 20–35 years [6]. Routine CRC screening is typically performed at older ages; therefore, the younger patients are often diagnosed at advanced stages. This scenario highlights an important gap in early CRC detection [6]. Most colorectal cancers gradually develop from precursor lesions (sessile serrated lesions and adenomatous polyps) over 10–15 years, providing a critical early detection window [7]. However, CRC remains asymptomatic in early stages and is often diagnosed at advanced stages after the onset of symptoms, leading to impaired clinical outcomes [8]. The development of CRC is summarized in Figure 1.

The rising occurrence of CRC coupled with limitations of current screening methods necessitates the development of novel non-invasive methods for early disease detection [13]. Invasiveness, limited detection sensitivity at early stages, and low patient adherence limit the effectiveness of currently available CRC screening programs [14]. The current study provides an overview of recent advances in cfDNA (cell-free DNA)-based technologies for CRC screening and early detection. Moreover, it evaluates their diagnostic performance and discusses current biological and technical challenges that could potentially impact the clinical implementation. The Saudi Arabian public-health context is discussed as an illustrative regional example of challenges relevant to the potential implementation of cfDNA-based screening.

## 2. Materials and Methods

### 2.1. Study Design

This narrative review summarizes recent literature on cfDNA-based blood tests for early colorectal cancer detection. The review evaluates the diagnostic efficacy of various cfDNA-based approaches [specificity, sensitivity, and AUC (area under the curve)] and compares the findings with established stool- and endoscopy-based screening methods.

### 2.2. Search Strategy

Screening of electronic databases (ScienceDirect, PubMed, and Google Scholar) assisted in identifying relevant literature. Only English-language articles, published between 2021 and 2025, were examined to capture recent advances in cfDNA-based technologies and CRC screening. The keywords “colorectal cancer”, “screening”, “cell-free DNA”, “cfDNA”, “liquid biopsy”, “blood-based tests”, “methylation”, “fragmentation”, “mutation analysis”, “copy number variation”, and “multi-omics” were used during the search process. Additional relevant studies were identified by screening the reference lists of the selected articles.

### 2.3. Study Selection

The selected studies encompassed human participants undergoing CRC screening or diagnostic evaluation. These studies assessed cfDNA-based assays or conventional CRC screening methods and reported early detection-related diagnostic performance outcomes [sensitivity, specificity, or AUC (area under the curve)]. Studies investigating advanced adenomas or advanced precancerous lesions, as defined in the original studies, were considered as well. The exclusion criteria included non-human studies, non-English publications, conference abstracts, letters, editorials, and studies not directly related to CRC screening or cfDNA-based detection. The initial database search, title and abstract screening, and full-text assessment were conducted by a primary authors, whereas the final study eligibility and data extraction were reviewed in consultation with co-authors possessing domain expertise.

### 2.4. Data Extraction

The parameters that were extracted from selected studies included study design, population characteristics, sample size, type of screening test, target biomarkers, and key diagnostic performance outcomes. Furthermore, stage-specific results related to early-stage CRC detection and additional information regarding clinical applicability and feasibility were retrieved as well, when available.

Terminology was retained as reported in the original studies. “Advanced adenoma” and “advanced precancerous lesion” were treated as study-specific categories rather than interchangeable terms, whereas “premalignant lesion” was used only as a general descriptive term.

Given the narrative scope of this review, differences in study design, populations, case–control enrichment, and validation settings were considered descriptively when interpreting the reported findings. A formal risk-of-bias assessment was not performed.

## 3. Results

### 3.1. Invasive Screening Methods

Invasive endoscopic methods, particularly colonoscopy and flexible sigmoidoscopy, are established CRC screening approaches. Colonoscopy has been associated with reductions in CRC incidence and mortality [15,16], but procedure completion and screening participation remain key limitations [16,17]. Flexible sigmoidoscopy reduces CRC incidence and mortality, particularly for distal colorectal lesions [18,19,20]. Table 1 provides a condensed overview of the included studies.

### 3.2. Non-Invasive Screening Methods

Non-invasive CRC screening strategies include FIT and FOBT, multitarget stool assays, and blood-based tests. FIT-based screening has been evaluated for cost-effectiveness [21]. A regional evaluation of the Finnish national screening program demonstrated that 49% of detected CRC cases were diagnosed at stage I [22]. Its clinical effectiveness depends on completing follow-up colonoscopy after a positive result [23,24]. FIT was preferred by many surveyed Saudi adults [25], whereas a separate study reported high hypothetical acceptance of annual blood-based surveillance [14].

Multitarget stool DNA and RNA assays reported higher sensitivity for CRC than FIT. The mt-sDNA study evaluated advanced precancerous lesions, whereas the mt-sRNA study investigated advanced adenomas. Both assays demonstrated higher sensitivity than FIT for their respective lesion categories, although their specificity was comparatively lower [26,27]. Real-world mt-sDNA data also showed high adherence to repeat screening and follow-up colonoscopy [28]. Table 2 provides a condensed overview of the included non-invasive screening studies.

### 3.3. cfDNA-Based Detection Approaches

Circulating cell-free DNA (cfDNA) is a non-invasive potential biomarker for CRC detection. It reflects tumor development-associated genetic and epigenetic alterations [30,31,32]. Several cfDNA analytical approaches have been investigated, including fragmentomics, integrated multi-omics strategies, mutation analysis, and methylation profiling.

#### 3.3.1. cfDNA Fragmentomics

AUC values ranged between 0.926 and 0.986 in three fragmentomics studies. These values were not statistically pooled, and direct comparison was limited by differences in study designs, populations, and analytical platforms [33,34,35]. In addition to sequencing-based approaches, cost-effective PCR-based assays have also been explored for assessing cfDNA fragmentation patterns of CRC [36]. Table 3 summarizes key studies evaluating cfDNA fragmentomics for CRC detection.

#### 3.3.2. cfDNA Methylation

AUC values ranged between 0.763 and 0.92 in four methylation studies. These values were not statistically pooled, and direct comparison was limited by differences in study design, population, and analytical platform [7,37,38,39]. Table 4 summarizes key studies evaluating cfDNA methylation biomarkers for CRC detection.

#### 3.3.3. cfDNA Mutation Analysis

Mutation-based cfDNA approaches illustrated varying diagnostic performance for CRC detection. AUC values of 0.80 to 0.89 have been reported in two studies. These values were not statistically pooled, and direct comparisons were limited by different study designs, populations, and analytical platforms [40,41]. Targeted sequencing and next-generation sequencing (NGS)-based assays identified recurrent mutations in various CRC-related genes (*FBXW7*, *TP53*, *APC*, *KRAS*, and *PIK3CA*) [40,41,42]. However, the diagnostic efficiency of mutation-based approaches remained generally limited in the early stages of disease and premalignant lesions. Circulating tumor DNA (ctDNA) is the tumor-derived fraction of cfDNA [43]. Its low abundance, particularly in early-stage CRC, could limit the detection of tumor-associated variants [40,42,43]. Table 5 summarizes key studies evaluating mutation-based cfDNA approaches for CRC detection.

#### 3.3.4. cfDNA Multi-Omics Approaches

Integrated cfDNA multi-omics approaches incorporate combinations of mutation profiling, methylation, fragmentomics, and copy number variation (CNV) for CRC detection. AUC values of 0.92 to 0.981 have been reported in two studies. These values were not statistically pooled, and direct comparison was limited by differential study design, population, and analytical platform [44,45]. Both estimates were derived from CRC-enriched cohorts rather than average-risk screening populations. In contrast, the large prospective ECLIPSE study evaluated the Shield cfDNA blood-based test in an average-risk screening population. It reported an 83.1% sensitivity for CRC, 89.6% specificity for advanced neoplasia, and only 13.2% sensitivity for advanced precancerous lesions [46]. Therefore, further optimization is necessary for detecting advanced precancerous lesions. Table 6 enlists the studies evaluating integrated cfDNA multi-omics approaches for CRC detection.

**Figure 2:**
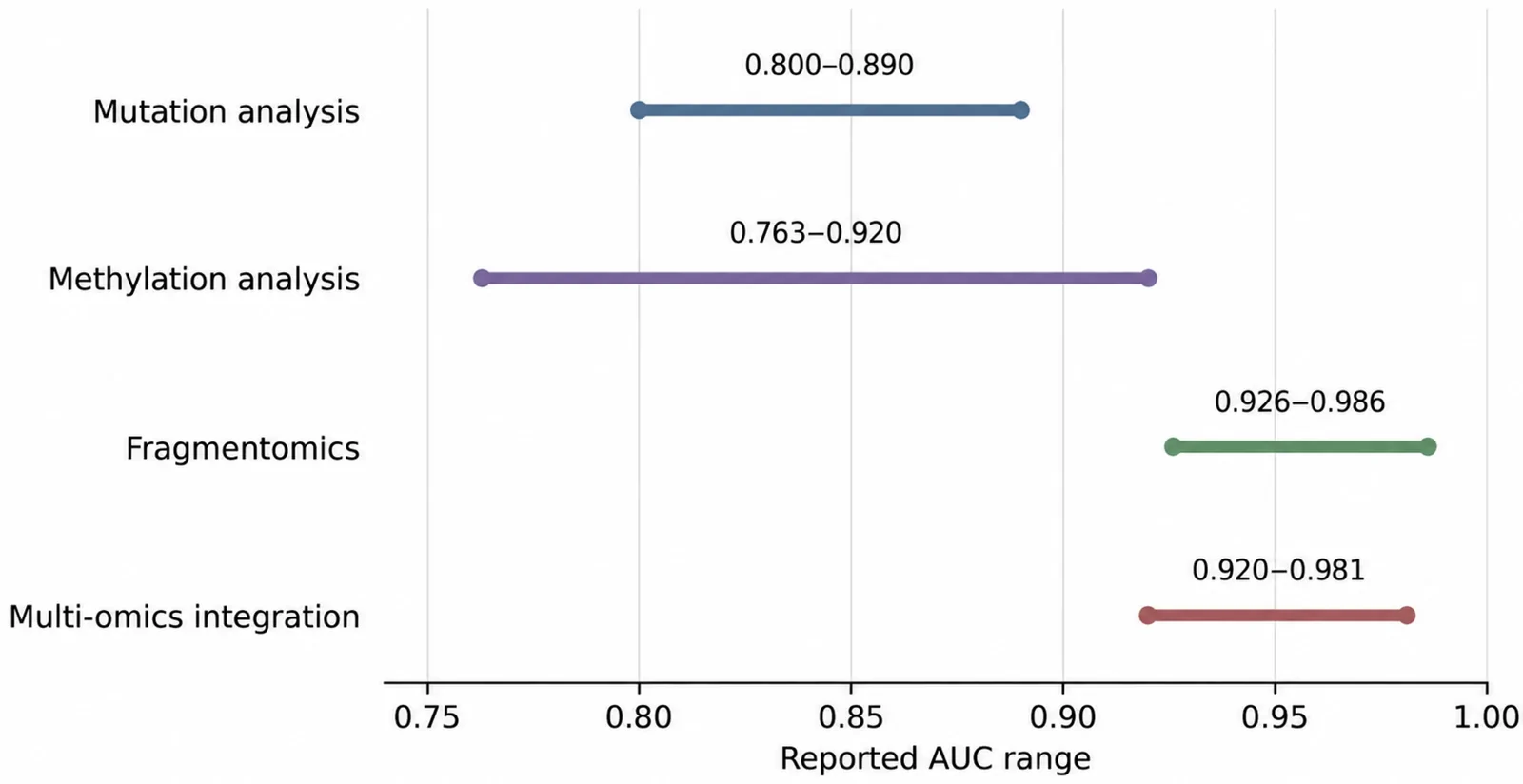
Descriptive presentation of individually reported AUC ranges for major cfDNA-based analytical approaches used in CRC detection. The displayed ranges were derived from heterogeneous studies summarized in Table 3, Table 4, Table 5 and Table 6. They do not represent pooled estimates or a ranking of the analytical approaches.

Overall, fragmentomics and integrated multi-omics approaches reported encouraging diagnostic performance in individual studies. However, differences in study design and population limited the direct comparison. Thus, it does not support a definitive ranking of these approaches (Figure 2).

## 4. Discussion

Colonoscopy is the leading invasive CRC screening method. It facilitates early cancer detection and removal of premalignant lesions to alleviate CRC incidence and mortality [47]. Its effectiveness depends on screening participation, procedural quality, follow-up, and implementation methods [15,16]. The modest benefits during the NordICC trial could be attributed to low screening uptake rather than the attenuated efficacy of colonoscopy [16]. Flexible sigmoidoscopy is another valuable alternative for distal CRC, particularly where colonoscopy resources are limited [18,19,20]. Despite appropriate referral practices, low public awareness and participation limit the screening outcomes in Saudi Arabia [16,48]. These findings highlight the role of organized screening programs to improve participation and optimize healthcare resources [49,50].

FIT serves as the first-line non-invasive screening test. The features like population acceptability, diagnostic performance, and cost-effectiveness favour its applicability [51]. Its effectiveness largely depends on timely colonoscopy after a positive result. Organized FIT-based programs generally improve early CRC detection; however, poor adherence to follow-up colonoscopy could reduce screening efficacy [22,24]. Saudi Arabian studies have reported similar challenges, where suboptimal screening uptake and follow-up hindered the effectiveness of FIT and FOBT [25,29]. Therefore, expanding public awareness and improving adherence could significantly increase FIT efficiency rather than further optimization [16,52].

Multitarget stool-based assays exhibited higher sensitivity for CRC than FIT [26,27]. Compared with FIT, mt-sDNA displayed higher sensitivity for advanced precancerous lesions [26], whereas mt-sRNA demonstrated higher sensitivity for advanced adenomas [27]. However, both assays reported lower specificity than FIT [26,27]. Advances in RNA-based assays and stool DNA methylation have further enhanced diagnostic efficiency, thus establishing molecular stool testing as an alternative CRC screening method [53,54,55]. Nevertheless, factors like limited accessibility, higher costs, and scarce real-world validation hinder their large-scale implementation [28,56]. Accordingly, these assays are considered complementary detection methods rather than replacement screening techniques [56,57].

Blood-based screening is another convenient CRC detection method for individuals unable/unwilling to undergo endoscopic or stool-based screening. This approach could improve screening participation [12,56]. However, recently developed assays are often less effective than conventional screening methods, particularly during the detection of advanced precancerous lesions [57]. Currently, blood-based tests serve as complementary strategies rather than replacement screening tools. Further advancement in molecular biomarkers could improve their clinical utility [56,57].

Fragmentomics has emerged as a promising cfDNA-based approach for CRC detection. It examines cfDNA fragmentation characteristics, such as fragment size, nucleosome positioning, end motifs, and alterations in copy number [33,34,35,36]. Whole-genome machine-learning models reported high diagnostic performance in individual studies [33,34,35,36]. However, their results should not be directly compared because the studies differed in design, populations, and validation methods [33,34,35,36]. A separate targeted ddPCR study evaluated cfDNA fragment sizes in three selected genomic regions but did not report diagnostic performance metrics [36].

Methodological differences (pre-analytical processing, sequencing platforms, and analytical workflows) substantially contributed to variability across examined studies [58,59,60]. Standardized bioinformatics and laboratory protocols are necessary for enhancing reproducibility and clinical translation [58,59,60]. Collectively, these findings support further evaluation of blood-based fragmentomics for CRC detection rather than establishing its screening performance, which necessitates large prospective validation studies [61,62].

The consistently favorable diagnostic performance across the included studies advocates cfDNA methylation as a promising biomarker for CRC detection. Aberrant DNA methylation during the early stage of colorectal carcinogenesis exhibits high tissue specificity [61]. The varying diagnostic efficiency in various studies could be attributed to different study populations, methylation targets, and analytical platforms rather than the biological value of the methylation [37,38,39]. Notably, studies on CRC-specific methylation panels have reported encouraging diagnostic efficiency. However, these studies highlighted the crucial role of appropriate biomarker selection in higher diagnostic accuracy [63,64].

Despite clinical potential, further optimization and validation of methylation-based assays is mandatory before their widespread clinical implementation. The integration of methylation and complementary epigenetic features could significantly enhance diagnostic accuracy and support the transition toward multimodal cfDNA analysis [65]. Standardized biomarker panels and large prospective validation studies are necessary for routine clinical implementation of these assays [61,63].

The diagnostic performance of mutation-based cfDNA assays remained generally lower than fragmentomic and methylation-based approaches, particularly during early-stage CRC and premalignant lesions’ detection [40,41,42]. This could be due to the low proportion of ctDNA within total cfDNA during early-stage disease, which restricts the detection of low-frequency variants [43]. Nevertheless, targeted sequencing panels enhanced the detection of recurrent CRC-associated mutations in *APC*, *PIK3CA*, *KRAS*, and *TP53*. Their overall diagnostic efficiency was limited by tumor heterogeneity and low ctDNA abundance [40,41,42].

Methodological differences led to diagnostic variations across examined studies. For example, targeted next-generation sequencing yielded higher sensitivity and better sequencing depth for low-frequency variants’ detection than whole-exome sequencing. These findings highlighted the crucial role of selecting appropriate analytical platforms for mutation analysis [42,43]. Overall, mutation analysis alone showed limited sensitivity for early CRC detection in the included studies. Therefore, it could be more effective in integration with complementary cfDNA biomarkers (fragmentomics and methylation) [43,61].

### 4.1. Clinical Challenges and Future Clinical Implementation

Despite the encouraging progress of cfDNA-based detection, the associated challenges require further in-depth investigations before its routine applications in CRC screening programs. Clinically, the procedural and systemic factors (comorbidities, surgery, and increasing age) could impact cfDNA concentrations and influence cfDNA-based test results [66].

Population-specific factors should also be taken into consideration. Hypothetically, genetic diversity influences the performance of mutation-based assays in the Saudi population, thus requiring direct validation [67]. Factors such as metabolic/inflammatory conditions, age, and smoking could also modify circulating cfDNA levels in the absence of cancer. These scenarios necessitate careful interpretation of biomarkers [68].

Blood-based CRC screening offers high patient acceptability that could improve their participation as compared to conventional approaches. In addition, cfDNA-based strategies could support the triage of FIT-positive individuals to alleviate unnecessary colonoscopy procedures [62].

SEPT9 and ColonAiQ are briefly discussed as examples of methylation-based blood assays undergoing clinical evaluation, rather than as part of the cross-study performance comparison [56,69,70]. However, large prospective validation studies are mandatory before the incorporation of these assays into national screening programs. Advancements in bioinformatics integration and artificial intelligence (AI) could further improve biomarker interpretation to elevate diagnostic performance [60,71,72,73].

### 4.2. Saudi Arabia as a Regional Implementation Example

Saudi Arabia is a regional example of challenges associated with CRC screening implementation. Organized screening programs reduce CRC incidence and mortality globally [74], yet nationwide screening remains limited in the Middle East [75]. CRC incidence has substantially increased in Saudi Arabia [76], which contradicts declining or stable trends in several high-income countries [77]. Despite the country’s contribution to CRC research, screening uptake remains low [78,79], with poor participation in fecal-based screening and colonoscopy [46]. Increasing early CRC onset [80,81], limited awareness, misconceptions, fear of colonoscopy, and insufficient physician recommendations represent additional barriers [82,83]. Established screening methods could therefore be complemented by emerging blood-based approaches [84], particularly given the reported prevalence of adenomatous polyps in Saudi cohorts [85].

## 5. Conclusions

cfDNA-based technologies are gaining popularity as promising non-invasive approaches for CRC detection. Fragmentomics and integrated multi-omics strategies reported encouraging diagnostic performance in individual studies; however, methodological heterogeneity prevents a definitive ranking of these approaches. Methylation-based assays also illustrated encouraging results. However, the efficacy of mutation-based approaches remained limited for early-stage CRC screening due to the low abundance of ctDNA. Methodological heterogeneity and the lack of large prospective validation studies were found to be the main limitations in routine clinical implementation of recent strategies. Biomarker selection, standardization of pre-analytical procedures, and analytical workflows emerged as key factors to improve reproducibility and facilitate clinical implementation. Currently, cfDNA-based assays could serve as a complementary approach along with established detection methods. The progress in multi-omics approaches combined with validation in diverse populations would further support the clinical implementation of cfDNA-based screening for early CRC detection.

## Figures and Tables

**Table 1:** Condensed overview of invasive CRC screening methods.

Screening Method	Evidence Base	Key Findings	Main Limitations
Colonoscopy	Saudi retrospective study [17], multicenter RCT [16], and pooled comparative analysis [15]	Neoplasia was detected in ~33.9% of patients, with 51.8% procedure completion [17]. NordICC reported an 18% reduction in CRC incidence after 10 years [16]. The pooled comparative analysis reported reductions of 30% in CRC incidence and 32% in CRC mortality [15].	Incomplete follow-up [17], low screening participation [16], and simulation-based analysis [15].
Flexible sigmoidoscopy	Meta-analysis [18], registry-based analysis [19], and long-term multicenter RCT [20]	CRC incidence was reduced by ~29–41%, particularly for distal CRC [18]. Approximately 58% of CRC cases were located within the reach of flexible sigmoidoscopy [19]. Once-only flexible sigmoidoscopy reduced CRC incidence by 24% and CRC mortality by 25% [20].	Evaluation was limited to the distal colon [18], observational design [19], and limited effect on proximal CRC [20].

**Table 2:** Condensed overview of non-invasive CRC screening methods.

Screening Method	Evidence Base	Key Findings	Main Limitations
FIT and FOBT	Decision model [21]; national and population-based screening studies [22,23]; follow-up study [24]; preference survey [25]; and primary-care record review [29].	Sequential FIT was more cost-effective than direct colonoscopy [21]. Among detected CRC cases, 49% were stage I [22]. HSgFOBT achieved 73% participation. Among HSgFOBT-positive participants who underwent colonoscopy, CRC and advanced adenomas were detected in 4.8% and 9.5%, respectively [23]. Only 47.6% of FIT-positive patients completed follow-up colonoscopy [24]. FIT was preferred by 41.7% of surveyed Saudi adults [25], while FOBT was feasible in primary care [29].	Model-dependent findings [21]; limited comparative data [22]; incomplete colonoscopy follow-up [23]; small COVID-19-era sample [24]; potential online-survey selection bias [25]; and single-center design with low colonoscopy adherence [29].
Blood-based testing preference	Cross-sectional survey of high-risk adults [14]	A total of 72.3% of participants reported being comfortable with a hypothetical surveillance protocol involving annual blood tests between 5-yearly colonoscopies [14].	The survey assessed a hypothetical surveillance scenario rather than actual screening performance [14].
Multitarget stool assays	Prospective multicenter mt-sDNA study [26], prospective phase III mt-sRNA trial [27], and population-based retrospective mt-sDNA cohort [28]	The mt-sDNA test achieved 93.9% sensitivity for CRC and 43.4% sensitivity for advanced precancerous lesions [26]. The mt-sRNA test achieved 94.4% sensitivity for CRC and 45.9% sensitivity for advanced adenomas [27]. After a negative mt-sDNA result, 81.0% underwent repeat screening within 5 years, while 84.4% completed colonoscopy within 1 year after a positive result [28].	Unevaluable samples and no direct comparison with the currently available mt-sDNA test [26], dropout related to the decentralized study design [27], and limited racial diversity [28].

**Table 3:** Detailed characteristics of cfDNA fragmentomics studies for colorectal cancer (CRC) detection.

Study (Author, Year, Ref.)	Sample Size & Population	Methodology/Platform	Fragmentomic Features	Performance Metrics	Key Limitations
Koval et al. (2023) [36]	85 participants (53 CRC, 32 healthy controls)	ddPCR-based targeted fragment size analysis	cfDNA fragment size patterns in open- and closed-chromatin regions	Not reported	Limited number of genomic loci analyzed; absence of tumor fraction estimation
Zhou et al. (2024) [35]	620 participants (healthy individuals, benign colorectal diseases, and CRC)	Low-pass WGS + LightGBM machine learning model	Fragment size profile and cfDNA end motifs	Sensitivity 96.2%, specificity 88.8%, AUC 0.95	Limited external validation; single-country population
Cao et al. (2024) [34]	838 participants (422 CRC, 416 healthy controls)	Low-pass WGS + ensemble machine learning model (FRAGTECT)	Fragment size ratio (FSR), fragment size distribution (FSD), copy number variation (CNV), nucleosome footprinting, and mutational context	Sensitivity 93.2%, specificity 97.5%, AUC 0.986	Single-center study; requires validation in larger and more diverse cohorts
Chen et al. (2025) [33]	394 participants (167 CRC, 227 non-cancer controls) from four hospitals	Low-pass WGS + stacked ensemble machine learning model	CNV, arm-level fragment size distribution (ARM-FSD), and mutation context/mutational signatures (MCMS)	Sensitivity 91.3%, specificity 82.3%, AUC 0.926	Retrospective case–control design; requires prospective validation in screening populations

Note: Metrics are reported as presented in the original studies. Sensitivity and specificity are percentages, while AUC ranges from 0 to 1. Fixed-specificity thresholds are stated where reported.

**Table 4:** Detailed characteristics of cfDNA methylation-based studies for colorectal cancer (CRC) detection.

Study (Author, Year, Ref.)	Sample Size & Population	Methodology/Platform	Methylation Targets	Performance Metrics	Key Limitations
Wang et al. (2024) [7]	1194 participants (398 CRC, 154 advanced adenomas, and 642 controls)	Quantitative methylation-specific PCR (qMSP)	NTMT1 and MAP3K14-AS1	Sensitivity 91.2%, specificity 92.4%, AUC 0.918	Requires prospective validation in asymptomatic screening populations
Long et al. (2024) [38]	169 participants (95 CRC and 74 healthy controls)	WGBS discovery followed by qMSP and ddPCR validation	DAB1, PPP2R5C, and FAM19A5	Sensitivity 64.2%, specificity 78.4%, AUC 0.763	Single-center study; small validation cohort; requires larger prospective validation
Lei et al. (2024) [37]	407 participants (70 CRC, 121 gastric cancer, and 216 non-cancer gastrointestinal controls)	Targeted cfDNA methylation sequencing + logistic regression model	Gastrointestinal cancer-specific CpG methylation signatures	Sensitivity 83.0%, specificity 81.5%, AUC 0.883	Symptomatic clinical cohort; requires validation in screening populations
Truong et al. (2025) [39]	61 participants (32 early-onset CRC and 29 non-CRC controls)	cfMeDIP coupled with multiplex digital PCR (cfMeDIP-dPCR)	SEPT9, KCNQ5, and C9orf50	KCNQ5 achieved AUC 0.92 with sensitivity 85% at 90% specificity	Pilot case–control study with a small cohort; requires validation in larger and more diverse populations

Note: Metrics are reported as presented in the original studies. Sensitivity and specificity are percentages, while AUC ranges from 0 to 1. Fixed-specificity thresholds are stated where reported.

**Table 5:** Detailed characteristics of cfDNA mutation-based studies for colorectal cancer (CRC) detection.

Study (Author, Year, Ref.)	Sample Size & Population	Methodology/Platform	Mutation Targets	Performance Metrics	Key Limitations
Kalmár et al. (2023) [42]	78 tissue samples (27 adenomas and 51 CRC) with matched plasma cfDNA samples from 17 adenoma and 33 CRC patients	Whole-exome sequencing (WES) with complementary targeted panel sequencing	*APC*, *KRAS*, *TP53*, *BRAF*, and other CRC-related genes	Targeted panel sequencing detected 12/20 (60%) tumor somatic variants in cfDNA, whereas WES detected 4/20 (20%) of the corresponding variants in the same patients.	Single-center study; limited sample size and low concordance between tissue and plasma WES
Bisarro et al. (2024) [40]	52 participants (8 healthy controls, 24 adenomas, and 20 CRC patients)	Targeted NGS using the Oncomine Colon cfDNA Assay	14 CRC-related genes, including *TP53*, *KRAS*, *APC*, *PIK3CA*, and *FBXW7*	Sensitivity 60%, specificity 100%, and AUC 0.80 for CRC detection	Small sample size; limited sensitivity for adenoma and early-stage lesion detection
Chen et al. (2024) [41]	163 participants (85 colorectal adenomas and 78 stage I CRC patients)	Targeted sequencing of a 451-gene panel combined with a random forest model	15-gene mutation panel, including *TP53*, *PIK3CA*, *KRAS*, *CNTNAP5*, *APC*, *GATA6*, *FBXW7*, and *NRAS*	AUC 0.89 in the discovery cohort and 0.83–0.85 in validation cohorts	Retrospective study; requires prospective validation in larger populations

Note: Metrics are reported as presented in the original studies. Sensitivity and specificity are percentages, while AUC ranges from 0 to 1. Fixed-specificity thresholds are stated where reported.

**Table 6:** Detailed characteristics of CNV-based and integrated cfDNA multi-omics studies for colorectal cancer (CRC) detection.

Study (Author, Year, Ref.)	Sample Size & Population	Methodology/Platform	Integrated Features	Performance Metrics	Key Limitations
Černoša et al. (2024) [44]	72 participants (36 CRC patients and 36 healthy controls)	Whole-genome enzymatic methyl-seq (EM-seq) combined with machine learning (LDA classifier)	Fragmentomics and chromosome 18 CNV analysis	Sensitivity 83%, specificity 92%, and AUC 0.92	Small CRC-enriched cohort; internal test-set evaluation without external validation; requires validation in larger independent cohorts.
Gao et al. (2024) [45]	373 participants (182 CRC patients and 191 healthy controls)	Mutation Capsule Plus (MCP) technology with targeted sequencing and shallow whole-genome sequencing (sWGS)	DNA methylation, 5′ end motifs, CNV, and gene mutations	AUC 0.981, sensitivity 92.1%, and specificity 94.7%	CRC-versus-healthy-control design; separate validation cohort; absence of precancerous lesions; no prospective average-risk screening validation
Chung et al. (2024) [46]	7861 average-risk screening participants	Prospective ECLIPSE study evaluating the Shield cfDNA blood-based test	Genomic alterations, methylation signatures, and fragmentomic patterns	Sensitivity 83.1%, specificity 89.6%, and sensitivity for advanced precancerous lesions 13.2%	Low sensitivity for advanced precancerous lesions

Note: Metrics are reported as presented in the original studies. Sensitivity and specificity are percentages, while AUC ranges from 0 to 1. Fixed-specificity thresholds are stated where reported.

## Data Availability

No new data were created or analyzed in this study. Data sharing is not applicable to this article.
